# Differential regulation of NMDA receptors by d-serine and glycine in mammalian spinal locomotor networks

**DOI:** 10.1152/jn.00810.2016

**Published:** 2017-02-15

**Authors:** David Acton, Gareth B. Miles

**Affiliations:** School of Psychology and Neuroscience, University of St Andrews, St Andrews, Fife, United Kingdom

**Keywords:** motor control, central pattern generator, spinal cord, neuromodulation

## Abstract

Activation of *N*-methyl-d-aspartate receptors (NMDARs) requires the binding of a coagonist, either d-serine or glycine, in addition to glutamate. Changes in occupancy of the coagonist binding site are proposed to modulate neural networks including those controlling swimming in frog tadpoles. Here, we characterize regulation of the NMDAR coagonist binding site in mammalian spinal locomotor networks. Blockade of NMDARs by d(−)-2-amino-5-phosphonopentanoic acid (d-APV) or 5,7-dichlorokynurenic acid reduced the frequency and amplitude of pharmacologically induced locomotor-related activity recorded from the ventral roots of spinal-cord preparations from neonatal mice. Furthermore, d-APV abolished synchronous activity induced by blockade of inhibitory transmission. These results demonstrate an important role for NMDARs in murine locomotor networks. Bath-applied d-serine enhanced the frequency of locomotor-related but not disinhibited bursting, indicating that coagonist binding sites are saturated during the latter but not the former mode of activity. Depletion of endogenous d-serine by d-amino acid oxidase or the serine-racemase inhibitor erythro-β-hydroxy-l-aspartic acid (HOAsp) increased the frequency of locomotor-related activity, whereas application of l-serine to enhance endogenous d-serine synthesis reduced burst frequency, suggesting a requirement for d-serine at a subset of synapses onto inhibitory interneurons. Consistent with this, HOAsp was ineffective during disinhibited activity. Bath-applied glycine (1–100 µM) failed to alter locomotor-related activity, whereas ALX 5407, a selective inhibitor of glycine transporter-1 (GlyT1), enhanced burst frequency, supporting a role for GlyT1 in NMDAR regulation. Together these findings indicate activity-dependent and synapse-specific regulation of the coagonist binding site within spinal locomotor networks, illustrating the importance of NMDAR regulation in shaping motor output.

**NEW & NOTEWORTHY** We provide evidence that NMDARs within murine spinal locomotor networks determine the frequency and amplitude of ongoing locomotor-related activity in vitro and that NMDARs are regulated by d-serine and glycine in a synapse-specific and activity-dependent manner. In addition, glycine transporter-1 is shown to be an important regulator of NMDARs during locomotor-related activity. These results show how excitatory transmission can be tuned to diversify the output repertoire of spinal locomotor networks in mammals.

## Abstract (precis)

We provide evidence that NMDARs within murine spinal locomotor networks determine the frequency and amplitude of ongoing locomotor-related activity in vitro and that NMDARs are regulated by d-serine and glycine in a synapse-specific and activity-dependent manner. In addition, glycine transporter-1 is shown to be an important regulator of NMDARs during locomotor-related activity. These results show how excitatory transmission can be tuned to diversify the output repertoire of spinal locomotor networks in mammals.

during locomotor behaviors, such as walking, running, and swimming, the coordination and timing of muscle activation are determined by central pattern generator (CPG) networks within the spinal cord (Kiehn 2016). In mammals, excitatory glutamatergic transmission controls the frequency and intensity of network output (Kiehn et al. 2008; Talpalar and Kiehn 2010) and contributes to gait selection (Bellardita and Kiehn; 2015Crone et al. 2009; Talpalar et al. 2013). Modulation of excitatory signaling therefore underlies behavioral responses to challenges presented by the external environment (Bellardita and Kiehn 2015; Grillner 2006; Orlovsky et al. 1999).

Endogenous glutamate acts via both *N*-methyl-d-aspartate (NMDA) receptors (NMDARs) and non-NMDARs, namely α-amino-3-hydroxy-5-methyl-4-isoxazolepropionic acid (AMPA) and kainate receptors, with blockade of either resulting in alterations to locomotor-related output in mammals (Beato et al. 1997; Cazalets et al. 1992; Douglas et al. 1993; Talpalar and Kiehn 2010; Whelan et al. 2000). In isolated rat spinal cord preparations, blockade of NMDARs results in either pronounced reductions in the frequency and amplitude of locomotor-related rhythmic activity or cessation of activity (Beato et al. 1997; Bracci et al. 1998; Cowley et al. 2005). By contrast, blockade of NMDARs has been reported to increase the frequency of locomotor-related activity in mice (Whelan et al. 2000). It is unclear whether these disparate findings reflect fundamental differences in glutamatergic signaling between species or methodological differences between studies.

Unlike non-NMDARs, canonical GluN1/GluN2 subunit-containing NMDARs require the binding of a coagonist in addition to glutamate for their activation (Clements and Westbrook 1991; Johnson and Ascher 1987; Kleckner and Dingledine 1988; Paoletti et al. 2013). In the brain, the endogenous coagonist may be either glycine or d-serine, with one or the other dominating at a given synapse (Henneberger et al. 2010, 2013; Kalbaugh et al. 2009; Le Bail et al. 2015; Li et al. 2009, 2013; Meunier et al. 2016; Mothet et al. 2000; Papouin et al. 2012). By comparison, little is known about coagonist identity in the spinal cord.

Concentrations of d-serine in the mammalian spinal cord are considerably lower than those of glycine, and much lower than those detected in the brain (Miyoshi et al. 2012; Schell et al. 1997; Sasabe et al. 2007; Thompson et al. 2012). On this basis, glycine was proposed as the exclusive coagonist of NMDARs in the spinal cord (Schell et al. 1997). However, beneficial effects of d-serine degradation in rodent pain studies (Lefèvre et al. 2015; Moon et al. 2015) and data implicating aberrant d-serine metabolism in the pathogenesis of amyotrophic lateral sclerosis (ALS) (Mitchell et al. 2010; Paul and de Belleroche 2014; Sasabe et al. 2007) highlight the importance of further investigation into the role of d-serine in spinal cord physiology.

Glycine availability at excitatory synapses is tightly regulated by glycine transporter 1 (GlyT1) (Berger et al. 1998; Bergeron et al. 1998; Supplisson and Bergman 1997). Blockade of GlyT1 enhances rhythmic activity in spinal locomotor networks in *Xenopus* tadpoles, implying a role for glycine transport in the regulation of swimming (Issberner and Sillar 2007). Inhibition of GlyT1 also facilitates NMDAR activation in spinal forelimb networks in rats (Shimomura et al. 2015); however, it is unknown whether GlyT1 regulates rhythmic locomotor-related activity in mammals, and evidence that endogenous glycine gates NMDARs in the spinal cord is lacking.

Occupancy of the coagonist binding site is proposed to be regulated in an activity-dependent manner, resulting in dynamic modulation of NMDAR activation (Kalbaugh et al. 2009; Li et al. 2009, 2013). An unsaturated coagonist binding site could permit the regulation of glutamatergic signaling by adjustments to coagonist availability (Berger et al. 1998; Li et al. 2009, 2013). It has been shown that the coagonist binding site is unsaturated in rat in vitro spinal cord preparations (Brugger et al. 1990; Shimomura et al. 2015) and within the spinal cords of *Xenopus* tadpoles during swimming (Issberner and Sillar 2007). These reports suggest that changes in coagonist availability could modulate excitatory transmission within mammalian spinal locomotor networks to regulate behavior.

Although the gating of NMDARs via the coagonist binding site has been proposed as an important mechanism for dynamic regulation of glutamatergic transmission in spinal locomotor networks of frog tadpoles, ultimately determining behavioral output (Issberner and Sillar 2007), its role in the production of locomotor-related activity in mammals has not been characterized. In this study, we examine coagonist regulation of NMDARs during network activity in spinal cord preparations from postnatal mice. We show that blockade of NMDARs reduces the frequency and amplitude of rhythmic activity, whereas increasing coagonist availability enhances network activity. Furthermore, we show that occupancy of the coagonist binding site varies with the intensity of network activity, implying activity-dependent regulation. We also provide evidence that endogenous glycine and d-serine regulate NMDARs in a synapse-specific manner and have opposing actions on the frequency of network output. Finally, we demonstrate that GlyT1 is a potent regulator of activity at excitatory synapses. Together, these findings reveal the importance of coagonist binding site regulation during behaviorally relevant activity in a mammalian network.

## METHODS

### 

#### Tissue preparation.

All procedures performed on animals were conducted in accordance with the UK Animals (Scientific Procedures) Act 1986 and were approved by the University of St Andrews Animal Welfare and Ethics Committee. Spinal cords were isolated from postnatal day (P)1–P4 C57BL/6 mice as previously described (Jiang et al. 1999). In summary, animals were killed by cervical dislocation, decapitated, and eviscerated, before being transferred to a dissection chamber containing artificial cerebrospinal fluid (aCSF; equilibrated with 95% oxygen, 5% carbon dioxide, ~4°C). Spinal cords were then isolated between midthoracic and upper sacral segments, and ventral and dorsal roots were trimmed.

#### Ventral root recordings.

Isolated spinal cords were pinned ventral-side up in a recording chamber perfused with aCSF (equilibrated with 95% oxygen, 5% carbon dioxide; room temperature) at 10 ml/min. Glass suction electrodes were attached to the first or second lumbar ventral roots (L_1_, L_2_) on each side of the spinal cord to record flexor-related activity. In some experiments a further suction electrode was attached to the fifth lumbar ventral root (L_5_) to record the corresponding extensor-related activity. Locomotor-related activity was evoked by bath application of 5-hydroxytryptamine (5-HT; 15 µM) and dopamine (DA; 50 µM) and was characterized by rhythmic bursting alternating contralaterally between upper ventral roots and ipsilaterally between upper ventral roots and L_5_. For disinhibited preparations (Bracci et al. 1996a; Witts et al. 2012), strychnine (1 µM) and picrotoxin (60 µM) were applied to evoke rhythmic bursting that was synchronous in all roots. In some experiments, d(−)-2-amino-5-phosphonopentanoic acid (d-APV; 50 µM) or erythro-β-hydroxy-l-aspartic acid (HOAsp; 400 µM) were bath applied at the onset of locomotor-related bursting. Any drugs present during the control period were also present during application of further drugs and during washout. In all experiments, stable rhythmic bursting was established over a period of ~1 h before the control period. Rhythmic bursting was considered stable when the frequency, amplitude, and duration of bursts were unchanged over several minutes. Data were amplified and filtered (band-pass filter 30–3,000 Hz, Qjin Design) and acquired at a sampling frequency of 6 kHz with a Digidata 1440A analog-digital converter and Axoscope software (Molecular Devices, Sunnyvale, CA). Custom-built amplifiers (Qjin Design) enabled simultaneous online rectification and integration (50-ms time constant) of raw signals.

#### Data analysis.

Data were analyzed offline with DataView software (courtesy of Dr W. J. Heitler, University of St Andrews). Ventral-root bursts were identified from rectified/integrated traces; peak-to-peak amplitudes and durations were then measured from the corresponding raw traces. Amplitude was measured as a noncalibrated unit and is presented here in arbitrary units. For clarity, data in time-course plots are reported normalized to control values; however, all statistical analyses were performed on raw data. For time-course plots, data were averaged across 1-min bins and normalized to a 10-min precontrol period to permit comparison between preparations. To assess instantaneous frequency and amplitude, statistical comparisons were performed on raw data averaged over 5-min periods or 10-min periods for disinhibited preparations. Data were analyzed with Student’s *t*-tests. Circular plots were used to assess phase relationship between bursts recorded from the left and right sides of the spinal cord (Crone et al. 2009; Kjaerulff and Kiehn 1996; Witts et al. 2012) (statistiXL software, Nedlands, WA, Australia). Data points represent the phase of locomotor-related bursts recorded from right ventral roots. The beginning of the locomotor cycle is defined as the onset of left ventral root activity and has a value of 0. A value of 0.5 corresponds to strict alternation between right and left bursts. Rayleigh’s test for uniformity was used to assess mean burst onset time (Crone et al. 2009; Kjaerulff and Kiehn 1996; Witts et al. 2012). Vector direction represents mean burst onset time, and vector length represents the concentration of data points around the mean. All statistical tests were performed in SPSS Statistics for Windows, version 21.0 (IBM, Armonk, NY) or Excel 2013 (Microsoft, Redmond, WA). *P* values < 0.05 were considered significant.

#### Solution, drug, and enzyme preparation.

The aCSF used for dissections and recordings contained (in mM) 127 NaCl, 26 NaHCO_3_, 10 glucose, 3 KCl, 2 CaCl, 1.25 NaH_2_PO_4_, and 1 MgCl_2_. 5,7-Dichlorokynurenic acid (DCKA) was supplied by Abcam (Cambridge, UK); 5-HT, d-amino acid oxidase from porcine kidney (DAAO), d-APV, d-serine, glycine, l-serine, NMDA, picrotoxin, and strychnine were supplied by Sigma-Aldrich (Poole, UK); *N*-[(3R)-3-([1,1′-biphenyl]-4-yloxy)-3-(4-fluorophenyl)propyl]-*N*-methylglycine hydrochloride (ALX 5407) was supplied by Tocris Bioscience (Bristol, UK); HOAsp was supplied by Wako Chemicals USA (Richmond, VA). ALX 5407 and picrotoxin were dissolved in DMSO, the concentration of which did not exceed 0.1% (vol/vol) in working solutions; DCKA and HOAsp were dissolved in 1 eq. NaOH; all other drugs and DAAO were dissolved in reverse-osmosis water.

## RESULTS

### 

#### NMDAR activation enhances the frequency and amplitude of pharmacologically induced locomotor-related activity.

**Fig. 1.**
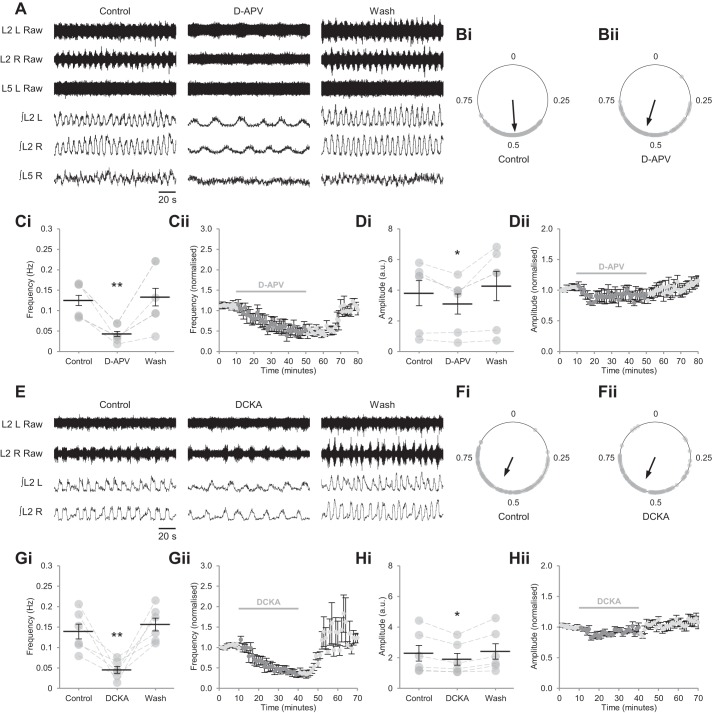
NMDARs determine the speed and amplitude of locomotor-related activity in spinal cord preparations from postnatal mice. *A*: raw (*top*) and rectified/integrated (*bottom*) traces recorded from the left and right L2 ventral roots (L2 L; L2 R) and right L5 ventral root (L5 R), showing the effect of the competitive glutamate-binding site antagonist d-APV (50 µM) on locomotor-related activity induced by 5-HT (15 µM) and DA (50 µM). *B*: left-right phase relationship in control conditions (*Bi*) and during application of d-APV (*Bii*). Circular plots represent the onset of locomotor bursts recorded from L2 R ventral roots (gray dots) in relation to the onset of activity recorded from corresponding L2 L roots (assigned a value of 0) in the same cycle. Vector direction indicates mean phase, and vector length corresponds to clustering of data points around the mean. We analyzed >100 burst cycles from 4 preparations for each condition. *Ci*: locomotor-burst frequency over 5 min during a control period, during a 40-min application of d-APV, and during a 30-min washout (Wash). Individual data points are shown in gray, and means are represented by black lines; *n* = 6 preparations. *Cii*: time-course plot of normalized data aggregated into 1-min bins showing a reduction in burst frequency during d-APV application; *n* = 6. *Di*: locomotor-burst amplitude over 5 min during a control period, during a 40-min application of d-APV, and during a 30-min washout; *n* = 6. *Dii*: time-course plot of normalized data aggregated into 1-min bins showing a reduction in burst amplitude during d-APV application; *n* = 6. *E*: raw (*top*) and rectified/integrated (*bottom*) traces recorded from L2 L and L2 R showing the effect of the competitive coagonist binding site antagonist DCKA (5 µM) on locomotor-related activity. *F*: left-right phase relationship in control conditions (*Fi*) and during application of DCKA (*Fii*). Circular plots represent the onset of locomotor bursts recorded from L2 R ventral roots (gray dots) in relation to the onset of activity recorded from corresponding L2 L roots (assigned a value of 0) in the same cycle. Vector direction indicates mean phase, and vector length corresponds to clustering of data points around the mean. We analyzed >100 burst cycles from 4 preparations for each condition. *Gi*: locomotor-burst frequency over 5 min during a control period, during a 30-min application of DCKA, and during a 30-min washout; *n* = 6. *Gii*: time-course plot of normalized data aggregated into 1-min bins showing a reduction in burst frequency during DCKA application; *n* = 6. *Hi*: locomotor-burst amplitude over 5 min during a control period, during a 30-min application of DCKA, and during a 30-min washout; *n* = 6. *Hii*: time-course plot of normalized data aggregated into 1-min bins showing a reduction in burst amplitude during DCKA application; *n* = 6. Error bars: ± SE. Statistically significant difference from control: *P < 0.05, **P < 0.01. a.u., Arbitrary units.

Reports conflict over the contribution of NMDARs to the production of fictive locomotion in mice (Nishimaru et al. 2000; Talpalar and Kiehn 2010; Whelan et al. 2000). To assess the role of NMDARs in isolated spinal cord preparations, we bath applied d-APV (50 µM), a selective, competitive NMDAR antagonist that interacts with the glutamate-binding site on GluN2 subunits, while recording pharmacologically induced (5-HT, 15 µM; DA, 50 µM), bilaterally alternating locomotor-related output from lumbar ventral roots. d-APV produced a marked reduction in the frequency of flexor-related bursting in L_2_ roots (64.9 ± 4.6%; *P* < 0.01, *n* = 6), from 0.125 ± 0.016 to 0.043 ± 0.011 Hz (Fig. 1, *A* and *C*). The amplitude of locomotor-related L_2_ bursts was also reduced, but to a lesser extent (16.7 ± 4.4%; Fig. 1, *A* and *D*; *P* < 0.05, *n* = 6). Alternation of bursts between contralateral L_2_ roots was maintained throughout the drug application (Fig. 1, *A* and *B*; Rayleigh’s test for uniformity: *P* < 0.001; >100 bursts from 4 preparations). Extensor-related activity recorded from L_5_ roots became indistinct upon application of d-APV (Fig. 1*A*) and was not further assessed. No differences were found between the effects of 50 and 100 µM d-APV on either the frequency (*P* > 0.05; *n* = 6) or amplitude (*P* > 0.05; *n* = 6) of bursting in L_2_ roots, indicating that receptors were saturated at the lower concentration, consistent with a previous report (Talpalar and Kiehn 2010).

To further investigate the contribution of NMDARs to locomotor-related activity, and to confirm the requirement for occupation of the coagonist binding site, we then applied DCKA (5 µM), a competitive inhibitor of NMDARs acting at the coagonist binding site on GluN1 subunits (Henderson et al. 1990). Like d-APV, DCKA potently reduced the frequency of locomotor-related bursting in L_2_ roots (63.7 ± 7.2%; Fig. 1, *E* and *G*; *P* < 0.01, *n* = 6) and modestly reduced burst amplitude (14.6 ± 2.6%; Fig. 1, *E* and *H*; *P* < 0.05, *n* = 6) without disrupting left-right alternation (Fig. 1, *E* and *F*; Rayleigh’s test for uniformity: *P* < 0.001; >100 bursts from 3 preparations). Together, these results indicate a role for NMDARs in controlling the frequency and amplitude of locomotor-related activity in mouse spinal cord preparations. Furthermore, these effects are mediated by canonical GluN1/GluN2 subunit-containing NMDARs (Pachernegg et al. 2012; Paoletti et al. 2013).

#### The NMDAR coagonist binding site is unsaturated during fictive locomotion.

**Fig. 2.**
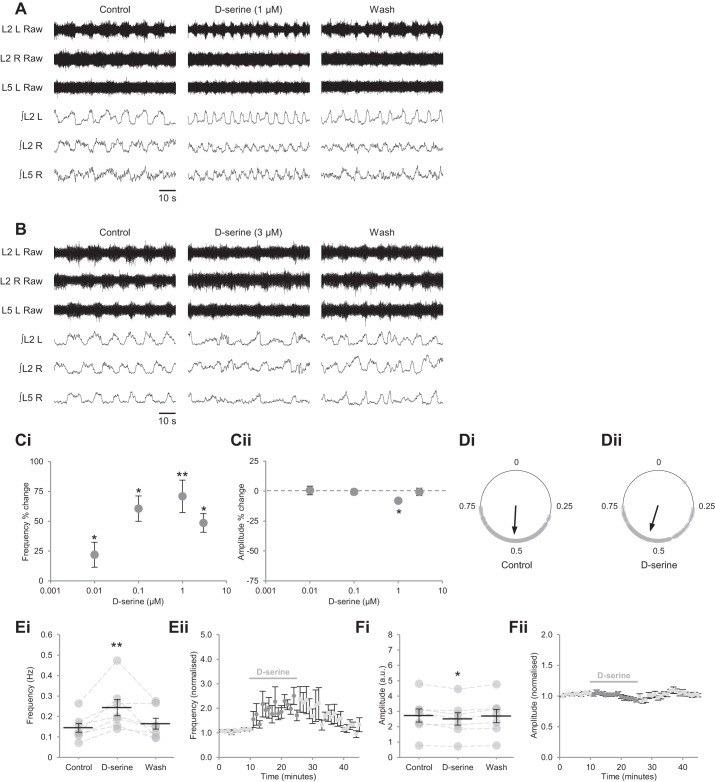
Exogenous d-serine modulates locomotor-related activity. *A* and *B*: raw (*top*) and rectified/integrated (*bottom*) traces recorded from the left and right L2 ventral roots (L2 L; L2 R) and right L5 ventral root (L5 R) showing the effects of d-serine at 1 µM (*A*) and 3 µM (*B*) on locomotor-related activity induced by 5-HT (15 µM) and DA (50 µM). *C*: percentage change in frequency (*Ci*) and amplitude (*Cii*) in response to varying concentrations of d-serine, calculated by comparing a 5-min window during a control period with a 5-min window during a 15-min application of d-serine; *n* = 5–7 preparations. *D*: left-right phase relationship in control conditions (*Di*) and during application of 1 µM d-serine (*Dii*). Circular plots represent the onset of locomotor bursts recorded from L2 R ventral roots (gray dots) in relation to the onset of activity recorded from corresponding L2 L roots (assigned a value of 0) in the same cycle. Vector direction indicates mean phase, and vector length corresponds to clustering of data points around the mean. We analyzed >100 burst cycles from 6 preparations for each condition. *Ei*: locomotor-burst frequency over 5 min during a control period, during a 15-min application of d-serine (1 µM), and during a 20-min washout. Individual data points are shown in gray, and means are represented by black lines; *n* = 7. *Eii*: time-course plot of normalized data aggregated into 1-min bins showing an increase in burst frequency during d-serine (1 µM) application; *n* = 7. *Fi*: locomotor-burst amplitude over 5 min during a control period, during a 15-min application of d-serine (1 µM), and during a 20-min washout; *n* = 7. *Fii*: time-course plot of normalized data aggregated into 1-min bins showing a reduction in burst amplitude during d-serine (1 µM) application; *n* = 7. Error bars: ± SE. Statistically significant difference from control: *P < 0.05, **P < 0.01.

**Fig. 3.**
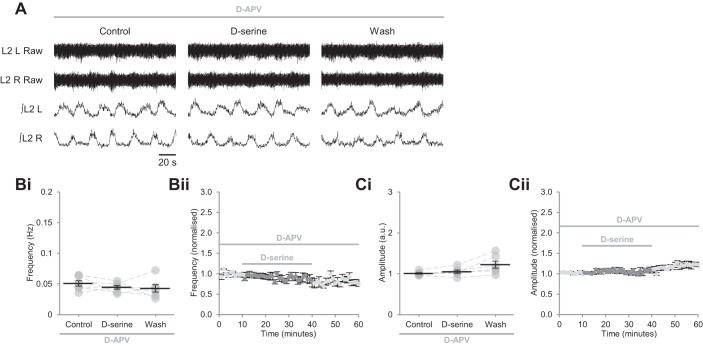
Bath-applied d-serine acts at NMDARs to modulate fictive locomotion. *A*: raw (*top*) and rectified/integrated (*bottom*) traces recorded from the left and right L2 ventral roots (L2 L; L2 R) showing the effect of d-serine (10 µM) on locomotor-related activity in the presence of the competitive glutamate-binding site antagonist d-APV (50 µM). *Bi*: locomotor-burst frequency over 5 min during a control period, during a 30-min application of d-serine (1–10 µM), and during a 20-min washout. d-APV was present throughout; *n* = 6. *Bii*: time-course plot of normalized data aggregated into 1-min bins showing no change in burst frequency when d-serine is applied in the presence of d-APV. *Ci*: locomotor-burst amplitude over 5 min during a control period, during a 30-min application of d-serine (1–10 µM), and during a 20-min washout. d-APV was present throughout. *Cii*: time-course plot of normalized data aggregated into 1-min bins showing no change in burst amplitude when d-serine is applied in the presence of d-APV; *n* = 6. Error bars: ± SE.

Although occupancy of the coagonist binding site by d-serine or glycine is a precondition of NMDAR activation (Clements and Westbrook 1991; Johnson and Ascher 1987; Kleckner and Dingledine 1988), coagonist binding sites within a given receptor population may not be saturated, in which case coagonist availability may be adjusted to modulate glutamatergic transmission during network activity (Berger et al. 1998; Kalbaugh et al. 2009; Li et al. 2009, 2013). We therefore sought to determine whether coagonist binding sites are saturated during fictive locomotion by bath applying d-serine at a range of concentrations (0.01 µM, *n* = 7 preparations; 0.1 µM, *n* = 5; 1 µM, *n* = 7; 3 µM, *n* = 6; duration: 15 min); d-serine, unlike glycine, is not subject to rapid clearance from the synapse (Supplisson and Bergman 1997). d-Serine increased burst frequency at concentrations of 0.01–3 µM within 1–2 min of application, with the greatest effect at 1 µM (70.9 ± 13.6%; Fig. 2, *A*, *Ci*, and *E*; *P* < 0.01, *n* = 7). At higher concentrations (Fig. 2, *B* and *Ci*), and when applied for periods longer than 15 min (not shown), rhythmic activity became disordered, and a smaller effect on burst frequency was recorded. d-Serine altered burst amplitude only at 1 µM, although the reduction detected at that concentration was modest (8.1 ± 2.1%; Fig. 2, *A*, *Cii*, and *F*; *P* < 0.05, *n* = 7). Alternation of bursts between contralateral L_2_ roots was maintained throughout the drug application (Fig. 2, *A* and *D*; Rayleigh’s test for uniformity: *P* < 0.001; >100 bursts from 6 preparations). Similarly, flexor-related bursting in L_5_ roots was maintained (Fig. 2*A*). To confirm that the effects of d-serine were mediated exclusively by NMDARs (Kakegawa et al. 2011), we applied it during NMDAR blockade. In the presence of saturating d-APV (50 µM), d-serine (1–10 µM) failed to modulate either the frequency (Fig. 3, *A* and *B*; *P* > 0.05, *n* = 6) or amplitude (Fig. 3, *A* and *C*; *P* > 0.05, *n* = 6) of rhythmic bursting. These data indicate that NMDARs within the spinal motor circuitry are unsaturated during fictive locomotion, permitting enhancement of NMDAR activation and thus network activity when coagonist concentration is increased.

#### Endogenous d-serine acts via NMDARs to reduce the frequency of locomotor-related activity.

**Fig. 4.**
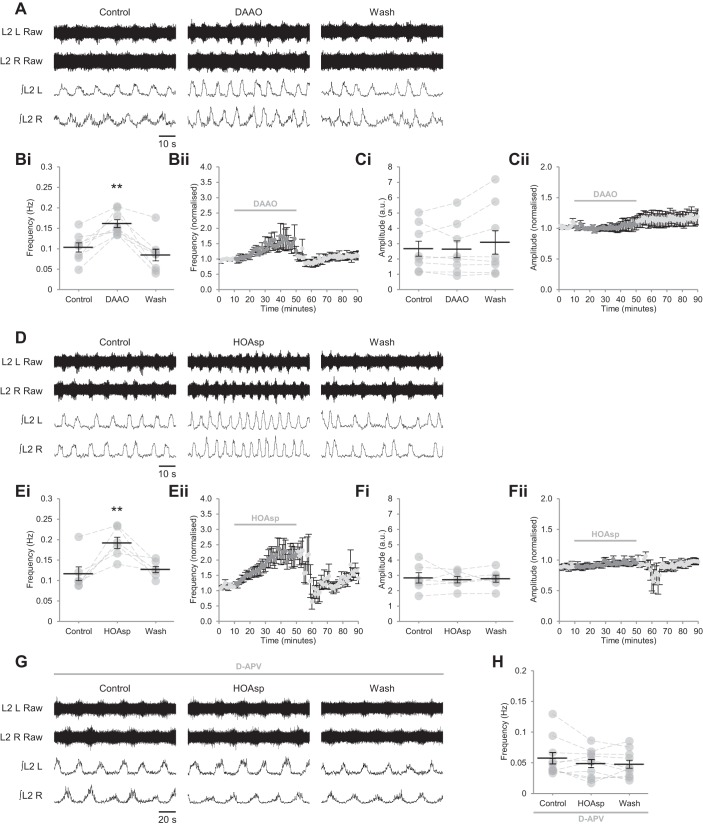
Endogenous d-serine acts via NMDARs to modulate the frequency but not the amplitude of locomotor-related activity. *A*: raw (*top*) and rectified/integrated (*bottom*) traces recorded from the left and right L2 ventral roots (L2 L; L2 R) showing the effect of the d-serine scavenger DAAO (0.29 U/ml) on locomotor-related activity induced by 5-HT (15 µM) and DA (50 µM). *Bi*: locomotor-burst frequency over 5 min during a control period, during a 40-min application of DAAO, and during a 40-min washout. Individual data points are shown in gray, and means are represented by black lines; *n* = 8. *Bii*: time-course plot of normalized data aggregated into 1-min bins showing an increase in burst frequency during DAAO application; *n* = 8. *Ci*: locomotor-burst amplitude over 5 min during a control period, during a 40-min application of DAAO, and during a 40-min washout; *n* = 8. *Cii*: time-course plot of normalized data aggregated into 1-min bins showing no change in burst amplitude during DAAO application; *n* = 8. *D*: raw (*top*) and rectified/integrated (*bottom*) traces recorded from L2 L and L2 R showing the effect of the serine-racemase inhibitor HOAsp (400 µM) on locomotor-related activity. *Ei*: locomotor-burst frequency over 5 min during a control period, during a 40-min application of HOAsp, and during a 40-min washout; *n* = 6. *Eii*: time-course plot of normalized data aggregated into 1-min bins showing an increase in burst frequency during HOAsp application; *n* = 6. *Fi*: locomotor-burst amplitude over 5 min during a control period, during a 40-min application of HOAsp, and during a 40-min washout; *n* = 6. *Fii*: time-course plot of normalized data aggregated into 1-min bins showing no change in burst amplitude during HOAsp application; *n* = 6. *G*: raw (*top*) and rectified/integrated (*bottom*) traces recorded from L2 L and L2 R showing the effect of HOAsp on locomotor-related activity in the presence of the competitive glutamate-binding site antagonist d-APV (50 µM). *H*: locomotor-burst frequency over 5 min during a control period, during a 30-min application of HOAsp, and during a 20-min washout. d-APV was present throughout; *n* = 10. Error bars: ± SE. Statistically significant difference from control: **P < 0.01.

In the brain, the endogenous NMDAR coagonist is either d-serine or glycine, with one or the other gating receptors in a nonredundant manner (Henneberger et al. 2010, 2013; Kalbaugh et al. 2009; Li et al. 2009, 2013; Le Bail et al. 2015; Meunier et al. 2016; Mothet et al. 2000; Papouin et al. 2012). We investigated coagonist identity within spinal networks by selectively depleting endogenous d-serine. Unexpectedly, selective degradation of endogenous d-serine by bath application of the enzyme DAAO (0.29 U/ml) (Papouin et al. 2012; Sasabe et al. 2014) resulted in a pronounced increase in the frequency of locomotor-related bursting (71.6 ± 20.7%; Fig. 4, *A* and *B*; *P* < 0.01, *n* = 8) but did not alter burst amplitude (Fig. 4, *A* and *C*; *P* > 0.05, *n* = 8). Likewise, application of HOAsp (400 µM) (Henneberger et al. 2010; Stříšovský et al. 2005) to inhibit endogenous serine racemase, which synthesizes d-serine from l-serine (Wolosker et al. 1999), increased the frequency of rhythmic bursting (77.9 ± 21.3%; Fig. 4, *D* and *E*; *P* < 0.01, *n* = 6) without affecting amplitude (Fig. 4, *D* and *F*; *P* > 0.05, *n* = 6). To confirm that the increase in locomotor frequency observed following depletion of endogenous d-serine was mediated by NMDARs, we bath applied HOAsp in the presence of d-APV (50 µM). In these experiments, neither burst frequency (Fig. 4, *G* and *H*; *P* > 0.05, *n* = 10) nor amplitude (*P* > 0.05, *n* = 10) were altered, indicating that endogenous d-serine acts at NMDARs. However, it is unlikely that endogenous d-serine acts at all available NMDARs because, if this were the case, its depletion would result in reduced frequency and amplitude of locomotor-related bursting, reproducing the effects of global NMDAR blockade. The finding that depletion of endogenous d-serine instead increases the frequency of network output implies that its action is restricted to a subset of NMDARs within pathways, likely involving inhibitory interneurons, that control the frequency of locomotor network output. Consistent with this hypothesis, it was recently shown that selective inhibition of a subset of glutamatergic neurons, proposed to synapse onto inhibitory spinal interneurons, enhances locomotor activity (Bouvier et al. 2015).

**Fig. 5.**
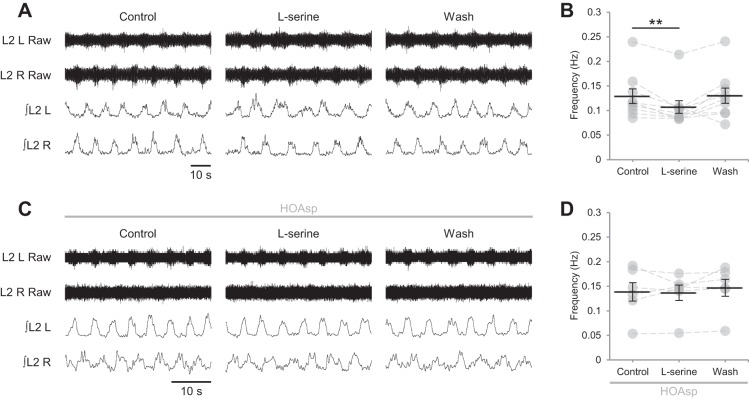
Racemization of l-serine within the spinal cord results in a decrease in the frequency of locomotor-related activity. *A*: raw (*top*) and rectified/integrated (*bottom*) traces recorded from the left and right L2 ventral roots (L2 L; L2 R) showing the effect of the d-serine precursor l-serine (50 µM) on locomotor-related activity induced by 5-HT (15 µM) and DA (50 µM). *B*: locomotor-burst frequency over 5 min during a control period, during a 15-min application of l-serine (40–100 µM), and during a 30-min washout. Individual data points are shown in gray, and means are represented by black lines; *n* = 9 preparations. *C*: raw (*top*) and rectified/integrated (*bottom*) traces recorded from L2 L and L2 R showing the effect of l-serine (50 µM) on locomotor-related activity in the presence of the serine-racemase inhibitor HOAsp (400 µM). *D*: locomotor-burst frequency over 5 min during a control period, during a 15-min application of l-serine (50 µM), and during a 30-min washout. HOAsp was present throughout; *n* = 6. Statistically significant difference from control: **P < 0.01.

To further investigate the activity of endogenous d-serine, experiments were performed in which the substrate of serine racemase, l-serine, was applied to preparations. Whereas bath-applied d-serine is expected to potentiate all NMDARs at which the coagonist binding site is unsaturated, application of l-serine is expected to enhance the availability of d-serine preferentially at synapses proximate to its synthesis (Rosenberg et al. 2010). The above experiments, in which DAAO and HOAsp were applied to reduce the actions of endogenous d-serine, suggest that d-serine is required at a subset of synapses, potentially onto inhibitory interneurons; l-serine application is therefore predicted to inhibit network output, having an effect opposite to that of DAAO and HOAsp. In agreement with this model, supplementation of l-serine (40–100 µM) consistently reduced the frequency of locomotor-related bursting 16.2 ± 3.6%; Fig. 5, *A* and *B*; *P* < 0.01, *n* = 9), without affecting amplitude (*P* > 0.05, *n* = 9). Burst frequency was unchanged when l-serine (50 µM) was applied in the presence of HOAsp (400 µM; Fig. 5, *C* and *D*; *P* > 0.05, *n* = 6), indicating that serine racemase is required for its conversion to d-serine, which in turn facilitates NMDAR activation. Collectively, these data support a role for endogenous d-serine in the regulation of a restricted population of NMDARs in the spinal cord, with glycine acting as the coagonist at the remaining fraction.

#### d-serine does not modulate excitatory components of locomotor networks during disinhibited bursting.

**Fig. 6.**
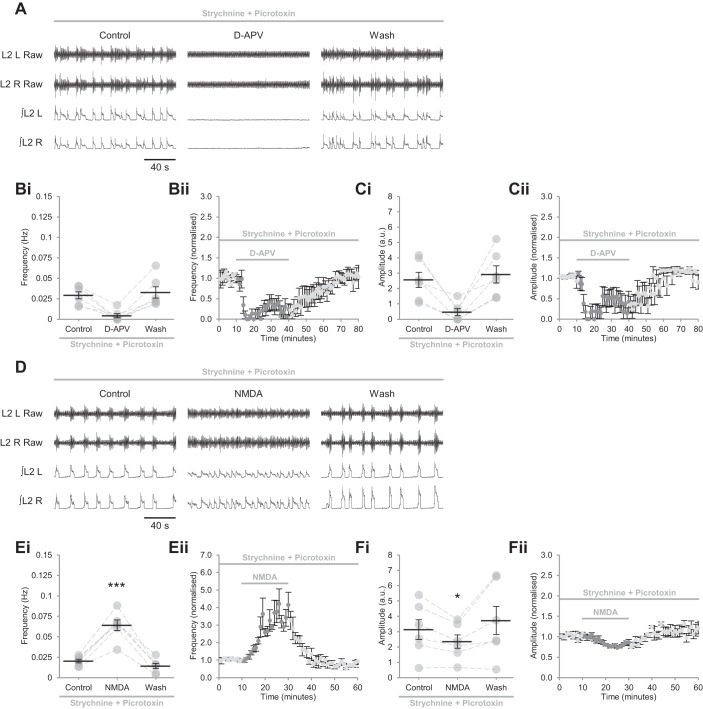
NMDA receptors are active during disinhibited bursting. *A*: raw (*top*) and rectified/integrated (*bottom*) traces recorded from the left and right L2 ventral roots (L2 L; L2 R) showing the effect of the competitive glutamate-binding site antagonist d-APV (50 µM) applied to preparations in which inhibitory transmission was blocked by the GABA_A_-receptor antagonist picrotoxin (60 μM) and the glycine-receptor antagonist strychnine (1 μM). *Bi*: ventral-root burst frequency over 10 min during a control period, during a 30-min application of d-APV, and during a 40-min washout. Individual data points are shown in gray, and means are represented by black lines; *n* = 6 preparations. *Bii*: time-course plot of normalized data aggregated into 1-min bins showing burst frequency during d-APV application; *n* = 6. *Ci*: ventral-root amplitude over 10 min during a control period, during a 30-min application of d-APV, and during a 40-min washout; *n* = 6. *Cii*: time-course plot of normalized data aggregated into 1-min bins showing burst amplitude during d-APV application; *n* = 6. *D*: raw (*top*) and rectified/integrated (*bottom*) traces recorded from L2 L and L2 R showing the effect of NMDA (10 µM) applied to preparations in which inhibitory transmission was blocked. *Ei*: ventral-root burst frequency over 10 min during a control period, during a 20-min application of NMDA, and during a 30-min washout; *n* = 6. *Eii*: time-course plot of normalized data aggregated into 1-min bins showing an increase in burst frequency during NMDA application; *n* = 6. *Fi*: ventral-root burst amplitude over 10 min during a control period, during a 20-min application of NMDA, and during a 30-min washout; *n* = 6. *Fii*: time-course plot of normalized data aggregated into 1-min bins showing a reduction in burst amplitude during NMDA application; *n* = 6. Error bars: ± SE. Statistically significant difference from control: *P < 0.05, ***P < 0.001.

**Fig. 7.**
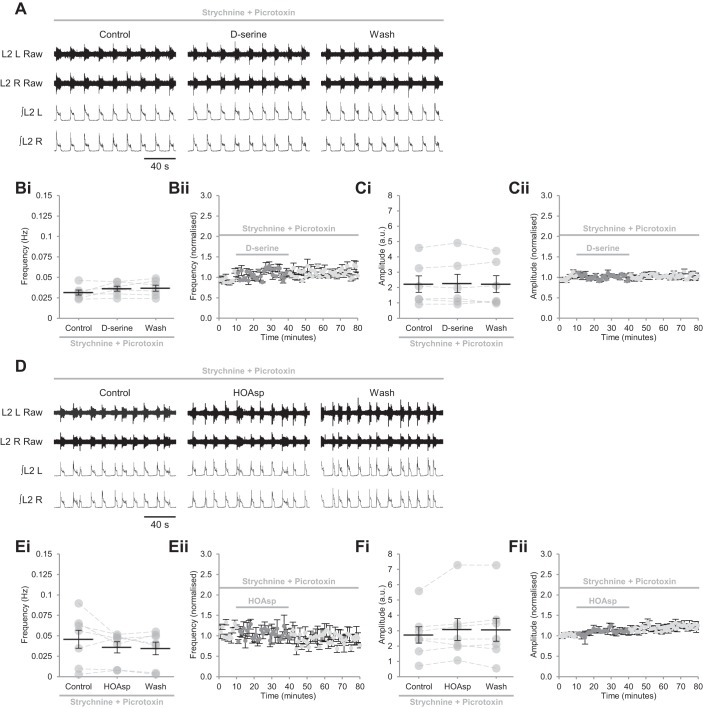
Neither exogenous nor endogenous d-serine modulates disinhibited activity mediated by excitatory components of locomotor networks. *A*: raw (*top*) and rectified/integrated (*bottom*) traces recorded from the left and right L2 ventral roots (L2 L; L2 R) showing the effect of d-serine (10 µM) applied to preparations in which inhibitory transmission was blocked by the GABA_A_-receptor antagonist picrotoxin (60 μM) and the glycine-receptor antagonist strychnine (1 μM). *Bi*: ventral-root burst frequency over 10 min during a control period, during a 30-min application of d-serine (1–10 µM), and during a 40-min washout. Individual data points are shown in gray, and means are represented by black lines; *n* = 6 preparations. *Bii*: time-course plot of normalized data aggregated into 1-min bins showing no change in burst frequency during d-serine application; *n* = 6. *Ci*: ventral-root burst amplitude over 10 min during a control period, during a 30-min application of d-serine (1–10 µM), and during a 40-min washout; *n* = 6. *Cii*: time-course plot of normalized data aggregated into 1-min bins showing no change in burst amplitude during d-serine application; *n* = 6. *D*: raw (*top*) and rectified/integrated (*bottom*) traces recorded from L2 L and L2 R showing the effect of the serine-racemase inhibitor HOAsp (400 µM) applied to preparations in which inhibitory transmission was blocked. *Ei*: ventral-root burst frequency over 10 min during a control period, during a 30-min application of HOAsp, and during a 40-min washout; *n* = 7. *Eii*: time-course plot of normalized data aggregated into 1-min bins showing no change in burst frequency during HOAsp application; *n* = 7. *Fi*: ventral-root burst amplitude over 10 min during a control period, during a 30-min application of HOAsp, and during a 40-min washout; *n* = 7. *Fii*: time-course plot of normalized data aggregated into 1-min bins showing no change in burst amplitude during HOAsp application; *n* = 7. Error bars: ± SE.

If endogenous d-serine acts as the dominant coagonist at a subset of NMDARs, it is likely that these are expressed by inhibitory interneurons that constrain the frequency of locomotor-related activity. To compare the sensitivity of excitatory and inhibitory components of the spinal motor circuitry to d-serine, we performed experiments in which inhibitory transmission was blocked by the glycine-receptor (GlyR) antagonist strychnine (1 μM) and the GABA_A_-channel antagonist picrotoxin (60 μM) (Bracci et al. 1996a; Foster et al. 2014; Witts et al. 2012). When isolated in this way, excitatory network components produced slow (0.032 ± 0.002 Hz, *n* = 25), long-duration, high-amplitude bursts that were synchronous across ventral roots (Figs. 6 and 7). When we applied d-APV (50 µM) to determine the importance of NMDARs for the generation of this pattern of network activity, bursting rapidly ceased in all experiments (Fig. 6, *A*, *Bii*, and *Cii*; *n* = 6), as previously reported in rats (Bracci et al. 1996a). In two of six experiments, however, bursting recovered during the period of d-APV application at a reduced frequency (Fig. 6*B*) and amplitude (Fig. 6*C*), indicating that NMDARs contribute to network excitation within disinhibited preparations, but are not indispensable. We also applied NMDA (10 µM) to confirm that NMDAR activation could be enhanced during disinhibited bursting. NMDA consistently increased burst frequency (221.2 ± 28.1%; Fig. 6, *D* and *E*; *P* < 0.001, *n* = 6), as previously reported in rats (Bracci et al. 1996b), while reducing burst amplitude (20.1 ± 5.0%; Fig. 6, *D* and *F*; *P* < 0.05, *n* = 6).

Next, we applied d-serine (1–10 µM) to assess whether NMDAR currents can be modulated via the coagonist binding site during disinhibited activity. Neither burst frequency (Fig. 7, *A* and *B*; *P* > 0.05, *n* = 6) nor amplitude (Fig. 7, *A* and *C*; *P* > 0.05, *n* = 6) were altered, indicating that NMDARs expressed by excitatory components of the locomotor circuitry have saturated coagonist binding sites under disinhibited conditions. To assess whether endogenous d-serine acts as an NMDAR coagonist within excitatory components of the locomotor circuitry, we applied HOAsp (400 µM) during disinhibited activity. Bursting was unchanged in both frequency (Fig. 7, *D* and *E*; *P* > 0.05, *n* = 7) and amplitude (Fig. 7, *D* and *F*; *P* > 0.05, *n* = 7) during HOAsp application, indicating that d-serine is not required as a coagonist by NMDAR populations involved in the production of disinhibited bursting. These results support differential regulation of spinal cord NMDARs via the coagonist binding site, whereby endogenous d-serine is required by NMDARs expressed by inhibitory components of the circuitry only, and receptors are saturated under some conditions but not others.

#### GlyT1 regulates extracellular glycine concentration and NMDAR activity.

**Fig. 8.**
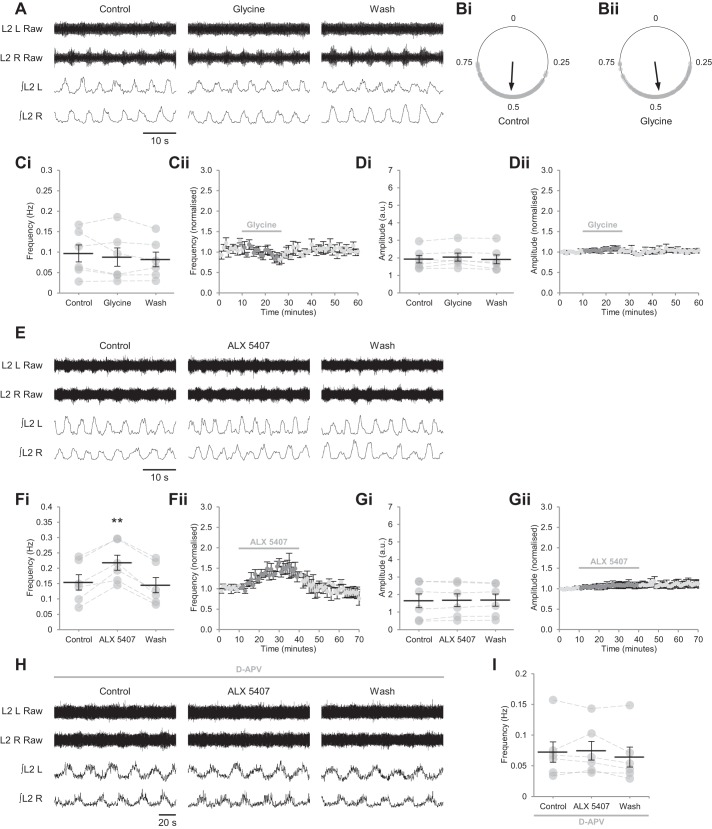
GlyT1 glycine transporters control the availability of glycine at excitatory synapses. *A*: raw (*top*) and rectified/integrated (*bottom*) traces recorded from the left and right L2 ventral roots (L2 L; L2 R) showing the effect of glycine (100 µM) on locomotor-related activity induced by 5-HT (15 µM) and DA (50 µM). *B*: left-right phase relationship in control conditions (*Bi*) and during application of 100 µM glycine 100 µM (*Bii*). Circular plots represent the onset of locomotor bursts recorded from L2 R ventral roots (gray dots) in relation to the onset of activity recorded from corresponding L2 L roots (assigned a value of 0) in the same cycle. Vector direction indicates mean phase, and vector length corresponds to clustering of data points around the mean. We analyzed >100 burst cycles from 3 preparations for each condition. *Ci*: locomotor-burst frequency over 5 min during a control period, during a 15-min application of glycine, and during a 40-min washout. Individual data points are shown in gray, and means are represented by black lines; *n* = 6. *Cii*: time-course plot of normalized data aggregated into 1-min bins showing no change in burst frequency during glycine application; *n* = 6. *Di*: locomotor-burst amplitude over 5 min during a control period, during a 15-min application of glycine, and during a 40-min washout; *n* = 6. *Dii*: time-course plot of normalized data aggregated into 1-min bins showing no change in burst amplitude during glycine application; *n* = 6. *E*: raw (*top*) and rectified/integrated (*bottom*) traces recorded from L2 L and L2 R showing the effect of the GlyT1 inhibitor ALX 5407 (30 µM) on locomotor-related activity. *Fi*: locomotor-burst frequency over 5 min during a control period, during a 30-min application of ALX 5407, and during a 30-min washout; *n* = 6. *Fii*: time-course plot of normalized data aggregated into 1-min bins showing an increase in burst frequency during ALX 5407 application; *n* = 6. *Gi*: locomotor-burst amplitude over 5 min during a control period, during a 30-min application of ALX 5407, and during a 30-min washout; *n* = 6. *Gii*: time-course plot of normalized data aggregated into 1-min bins showing no change in burst amplitude during ALX 5407 application; *n* = 6. *H*: raw (*top*) and rectified/integrated (*bottom*) traces recorded from L2 L and L2 R showing the effect of ALX 5407 on locomotor-related activity in the presence of the competitive glutamate-binding site antagonist d-APV (50 µM). *I*: locomotor-burst frequency over 5 min during a control period, during a 30-min application of HOAsp, and during a 30-min washout. d-APV was present throughout; *n* = 6. Error bars: ± SE. Statistically significant difference from control: **P < 0.01.

Because the action of endogenous d-serine appears limited to a subpopulation of NMDARs within spinal networks, we next considered the role of glycine in the gating of NMDARs during bilaterally alternating locomotor-related activity. Although glycine is the principal inhibitory transmitter within the spinal cord (Bowery and Smart 2006) and has a similar binding affinity and potency to d-serine at NMDAR coagonist binding sites (Brugger et al. 1990; Chen et al. 2008; Priestley et al. 1995), application of glycine did not modulate the frequency or amplitude of rhythmic activity at 1 µM (frequency: *P* > 0.05, *n* = 6; amplitude: *P* > 0.05, *n* = 6), 10 µM (frequency: *P* > 0.05, *n* = 6; amplitude: *P* > 0.05, *n* = 6) or 100 µM (frequency: *P* > 0.05, *n* = 6; amplitude: *P* > 0.05, *n* = 6; Fig. 8, *A*, *C*, and *D*), nor was left-right alternation of bursting altered (Fig. 8, *A* and *B*; Rayleigh’s test for uniformity: *P* < 0.001; >100 bursts from 3 preparations).

The glycine transporter GlyT1 is highly expressed at excitatory synapses in both the spinal cord and brainstem (Cubelos et al. 2005; Zafra et al. 1995b) and has been proposed to maintain sub-saturating concentrations of glycine in the latter (Berger et al. 1998; Lim et al. 2004). To determine the influence of GlyT1 on extracellular glycine and locomotor-related network activity, we applied the selective GlyT1 antagonist ALX 5407 (30 µM) (Atkinson et al. 2001). GlyT1 antagonism resulted in a gradual increase in burst frequency (52.0 ± 13.9%; Fig. 8, *E* and *F*; *P* < 0.01, *n* = 6) but had no effect on amplitude (Fig. 8, *E* and *G*; *P* > 0.05, *n* = 6). By contrast, no changes in locomotor frequency (Fig. 8, *H* and *I*; *P* > 0.05, *n* = 6) or amplitude (*P* > 0.05, *n* = 6) were observed when ALX 5407 (30 µM) was applied in the presence of d-APV (50 µM). Together, these results indicate that GlyT1 governs occupation of NMDAR coagonist binding sites by exercising stringent control over the concentration of glycine at excitatory synapses.

## DISCUSSION

We provide evidence that both endogenous d-serine and glycine gate NMDARs within mammalian spinal networks to regulate the frequency of locomotor-related activity, that the NMDAR coagonist binding site at which they act is unsaturated during locomotor-related but saturated during disinhibited activity, and that the glycine transporter GlyT1 is a potent regulator of glycine concentration at excitatory synapses. These findings suggest that dynamic regulation of NMDARs via the coagonist binding site extends the output repertoire of mammalian spinal motor circuits, thereby facilitating appropriate behavior.

Excitatory glutamatergic transmission is fundamental to the operation of spinal networks in both mammalian and nonmammalian vertebrates (Alford and Grillner 1990; Brodin et al. 1985; Cazalets et al. 1992; Dale and Roberts 1985; Douglas et al. 1993; Fenaux et al. 1991; MacDermott and Dale 1987). In mammals, glutamatergic transmission operates within spinal locomotor networks to determine the frequency, amplitude, and robustness of rhythmic activity (Talpalar and Kiehn 2010). In addition, glutamate released onto spinal networks from descending pathways mediates commands for both the initiation (Hägglund et al. 2010; Jordan et al. 2008) and cessation (Bouvier et al. 2015) of locomotion. Glutamatergic signaling within spinal networks is mediated by both NMDARs and non-NMDARs (Beato et al. 1997; Cazalets et al. 1992; Nishimaru et al. 2000; Talpalar and Kiehn 2010; Whelan et al. 2000). Whereas a role for NMDARs in the operation of spinal CPGs has been established in lampreys and tadpoles (Alford and Grillner 1990; Brodin et al. 1985; Dale and Roberts 1985; MacDermott and Dale 1987; Wallén and Grillner 1987), their importance for the generation of patterned activity in mammalian networks is less clear. Previously, it was proposed that the contribution of NMDARs to network output differs between isolated spinal cord preparations from postnatal rats and mice (Nishimaru et al. 2000). In rats, application of NMDAR agonists alone is reported to induce sustained fictive locomotion (Kudo and Yamada 1987; Smith et al. 1988), whereas in mice sustained activity is proposed to require 5-HT in addition to NMDAR agonists (Jiang et al. 1999). In addition, NMDAR blockade consistently inhibits lumbar rhythmic activity in rats (Beato et al. 1997; Bracci et al. 1998; Cazalets et al. 1992; Cowley et al. 2005; Gabbay and Lev-Tov 2004; Smith et al. 1988), whereas in mice, it has been reported to enhance burst frequency during pharmacologically induced (5-HT, 15 µM; DA, 75 µM) fictive locomotion (Whelan et al. 2000). We find that NMDAR blockade reduces the frequency of fictive locomotion in mouse spinal-cord preparations, as previously reported in rat preparations (Beato et al. 1997; Gabbay and Lev-Tov 2004; Cowley et al. 2005). The reason for the discrepancy between our results and those of Whelan et al. (2000) is unclear, since pharmacological techniques and recording conditions were similar between the two studies, and it would be surprising if the use of different mouse strains (Swiss Webster vs. C57BL/6) accounted for differences in a fundamental signaling mechanism.

We also report that NMDAR blockade reduces burst amplitude, an observation previously reported in mice (Whelan et al. 2000) and rats (Beato et al. 1997). As in rat preparations (Beato et al. 1997; Bracci et al. 1998; Cowley et al. 2005), left-right rhythmic alternation of bursts is maintained during NMDAR blockade. We do not consider the effects of NMDAR blockade at high frequencies (>0.4 Hz) of fictive locomotion, during which it has been reported to disrupt patterned output by increasing nonresetting deletions, rather than alter frequency per se (Talpalar and Kiehn 2010). Our data are consistent with previous reports that NMDARs are not essential for locomotor-related activity in rodents but instead function to regulate its amplitude and pacing, at least at lower frequencies (< 0.4 Hz) (Beato et al. 1997; Bracci et al. 1998; Cowley et al. 2005; Talpalar and Kiehn 2010). d-APV, a competitive inhibitor of the glutamate-binding site, and DCKA, a competitive inhibitor of the coagonist binding site, have similar effects on burst frequency and amplitude, revealing a requirement for binding of both glutamate and a coagonist, and implying that canonical GluN1/GluN2 subunit-containing NMDARs dominate in the spinal motor circuitry (Pachernegg et al. 2012; Paoletti et al. 2013).

The finding that extensor-related bursting recorded from L_5_ roots becomes indistinct upon NMDAR blockade may reflect relatively weak locomotor drive to the lower lumbar segments resulting from a rostrocaudal gradient of excitability within the locomotor circuitry (Bonnot et al. 2002). Consistent with this, NMDAR activation is not essential for bursting in L_5_, since L_5_ bursting continues during NMDAR blockade when a strong pharmacological stimulus is applied (Talpalar and Kiehn 2010). However, it cannot be ruled out that NMDAR activation is more important for the generation of activity in L_5_ roots compared with upper lumbar roots. The potential for differential excitatory coupling between network modules at different segmental levels (Hägglund et al. 2013) remains to be investigated.

We find that blockade of NMDARs results in sustained abolition of disinhibited bursting in most but not all preparations. Similarly, it is reported that NMDAR blockade abolishes disinhibited bursting in rat preparations, except when AMPA receptor currents are enhanced (Bracci et al. 1996a). Thus NMDARs make a substantial contribution to disinhibited activity in both rats and mice but are not essential for it. As in bilaterally alternating locomotor-related activity (Beato et al. 1997; Bracci et al. 1998; Cowley et al. 2005; Gabbay and Lev-Tov 2004; Nishimaru et al. 2000; Talpalar and Kiehn 2010; Whelan et al. 2000), disinhibited activity can be sustained during NMDAR blockade when an alternative stimulus of sufficient intensity is provided. Together, our results confirm the importance of NMDARs during both locomotor-related and disinhibited activity in mice, suggesting that NMDARs function in a similar manner in both mice and rats, by contrast with previous reports (Nishimaru et al. 2000; Whelan et al. 2000).

The coagonist binding site is reported to be unsaturated in some preparations (Berger, Dieudonné and Ascher 1998; Kalbaughet al. 2009; Le Bail et al. 2015; Li et al. 2009, 2013), but saturated in others (Li et al. 2009; Shigetomi et al. 2013), and activity-dependent changes in occupancy of the coagonist binding site have been reported in the brain (Kalbaugh et al. 2009; Li et al. 2009, 2013). We show that the NMDAR coagonist binding site is unsaturated during locomotor-related activity and may be subject to cell type-specific and activity-dependent regulation. An unsaturated coagonist binding site would permit the regulation of glutamatergic signaling and locomotor frequency by adjustments to coagonist availability (Berger et al. 1998; Li et al. 2009, 2013). We show that exogenous d-serine acts at NMDARs to increase the frequency of locomotor-related activity in a dose-dependent manner. The effective concentrations of exogenous d-serine are consistent with the binding affinities and potencies reported for d-serine and glycine, with EC_50_ values in the range of ~0.1–1.3 µM (Brugger et al. 1990; Chen et al. 2008; Priestley et al. 1995). However, bursting becomes disrupted at higher concentrations of d-serine, as previously reported for NMDA itself (Talpalar and Kiehn 2010), illustrating a requirement for regulation of NMDARs to ensure correct network activity.

The effect of bath-applied d-serine varies with the mode of network activity. Despite strong activation of NMDARs during disinhibited bursting, exogenous d-serine does not modulate this mode of activity, indicating that the coagonist binding sites of NMDARs expressed by the cells that generate it are saturated. Although it is uncertain whether populations of rhythmogenic interneurons are identical between disinhibited and locomotor-related preparations, the requirements for glutamatergic signaling and commissural circuit elements during synchronous disinhibited bursting (Bracci et al. 1996b; Talpalar et al. 2011) support a role for excitatory interneurons in the generation of this mode of activity, and these cells are proposed to form a rhythmogenic core common to disinhibited and locomotor-related activity (Bracci et al. 1996b). Thus excitatory interneurons likely mediate changes in the frequency of disinhibited bursting, including the frequency increase observed upon NMDA application, as they do during fictive locomotion (Kiehn et al. 2008; Kiehn 2016). An alternative possibility is that NMDA acts directly on motoneuronal NMDARs to increase the frequency of disinhibited bursting. However, since the coagonist binding site of motoneuronal NMDARs is not saturated during network quiescence (Brugger et al. 1990), an activity-dependent increase in coagonist availability is also implied in this scenario. Our data therefore imply differential availability of the coagonist depending on network activity. This may relate to stronger activation of glutamate receptors during disinhibited bursting, which is characterized by high-amplitude, long-duration bursts. Consistent with this possibility, it is reported that coagonist availability scales with glutamate release in brain preparations, providing positive feedback onto excitatory synapses (Li et al. 2009, 2013).

Whereas NMDA and glutamate enhance burst amplitude during locomotor-related activity at lower concentrations and reduce it at higher concentrations (Talpalar and Kiehn 2010), exogenous d-serine only modestly reduces burst amplitude. Since this effect occurs only at the concentration of d-serine that most potently increases burst frequency, it may be an indirect effect of rapid network activity. The failure of d-serine to enhance burst amplitude at lower doses implies that motoneuronal NMDARs are saturated during locomotor-related activity. Consistent with this possibility, burst amplitude is also unchanged when glycine availability is enhanced by blockade of GlyT1. Amplitude is also unaffected by d-serine application during disinhibited bursting, although this may be a function of more intense glutamate release (see above). By contrast, the coagonist binding site of motoneuronal NMDARs is unsaturated when network activity is silenced by TTX (Brugger et al. 1990). Thus the coagonist site may be subject to cell type-specific as well as activity-dependent regulation, implying considerable flexibility in the regulation of coagonist availability, serving to extend the range of possible network outputs.

In addition to activity-dependent regulation of coagonist availability, we provide evidence that the identity of the dominant coagonist differs between populations of NMDARs, with endogenous d-serine and glycine having distinct effects on network output. Whereas endogenous d-serine depresses the frequency of locomotor-related activity, glycine enhances the frequency of activity. If d-serine were the exclusive coagonist in spinal locomotor networks, or if it acted in conjunction with glycine at all NMDARs, its depletion would mimic the effects of NMDAR inhibition by d-APV or DCKA; that is, it would reduce the frequency and amplitude of locomotor-related activity. Instead, depletion of endogenous d-serine, either with an enzymatic scavenger or by inhibition of its synthesizing enzyme, results in a pronounced increase in burst frequency with no change in burst amplitude. Conversely, supplementation of the d-serine precursor l-serine results in a decrease in burst frequency that is dependent on d-serine synthesis. Exogenous l-serine presumably enhance d-serine production at synapses where serine racemase is concentrated and d-serine is utilized, whereas exogenous d-serine potentiates NMDARs at all synapses where the coagonist binding site is unsaturated. The finding that endogenous d-serine inhibits locomotor-related activity suggests that it is primarily required at excitatory synapses onto inhibitory interneurons. Consistent with this possibility, serine racemase inhibition has no effect when excitatory components of the network are examined in isolation. As a similar example of excitatory signaling acting to reduce network activity, glutamatergic signaling onto spinal cord networks by descending glutamatergic V2a interneurons mediates inhibition of locomotor-related activity, likely through excitation of inhibitory interneurons (Bouvier et al. 2015). By contrast, signaling onto the spinal cord network by a broader population of glutamatergic interneurons stimulates network activity, masking the influence of V2a cells (Hägglund et al. 2010). A further example of burst frequency regulation by inhibitory interneurons is provided by V1 cells, ablation of which reduces burst frequency (Gosgnach et al. 2006). Together, our observations suggest a model in which d-serine acts via a subpopulation of NMDARs expressed by inhibitory interneurons to constrain locomotor speed, with the remaining moiety of NMDARs being regulated by glycine. These results are consistent with studies showing differential regulation of distinct populations of NMDARs by glycine and d-serine in the brain, where the identity of the dominant coagonist varies with receptor subunit composition (Kalbaugh et al. 2009; Le Bail et al. 2015; Papouin et al. 2012) subcellular localization of NMDARs (Papouin et al. 2012), and activity (Li et al. 2013), as well as with developmental stage (Le Bail et al. 2015).

Although glycine acts at NMDARs with a similar affinity and potency to d-serine (Brugger et al. 1990; Chen et al. 2008; Priestley et al. 1995), which exerts a pronounced effect on the frequency of locomotor-related activity via NMDARs at 1 µM, exogenous glycine does not perturb locomotor-related activity at concentrations up to 100 µM. Although we cannot eliminate the possibility that glycine activity at strychnine-sensitive inhibitory GlyRs masks opposing activity at NMDARs, it is unlikely that GlyRs were activated in these experiments, as glycine has EC_50_ values two to three orders of magnitude higher for GlyRs than for NMDARs (Blednov et al. 1996; Engblom and Akerman 1991; Lewis et al. 1998; Tapia and Aguayo 1998; Graham et al. 2011; Young and Snyder 1974). Furthermore, inhibitory effects of glycine are not observed when the concentration of extracellular glycine is increased by GlyT1 blockade in the presence of d-APV. The lack of effect of exogenous glycine in our preparations parallels findings in the brainstem, where d-serine facilitates NMDAR currents in the low-micromolar range, but glycine is ineffective at concentrations below 100 µM unless GlyT1 is inhibited (Berger et al. 1998). The finding that exogenous glycine lacks effect is thus likely attributable to powerful glycine clearance by GlyT1, which is highly expressed in both the spinal cord and brainstem (Cubelos et al. 2005). GlyT1 is a potent regulator of glycine availability at excitatory synapses and is capable of holding glycine at subsaturating concentrations within the restricted space of the synaptic cleft, even when its concentration in the surrounding medium is an order of magnitude greater (Bergeron et al. 1998; Supplisson and Bergman 1997). Consistent with tight regulation of glycine concentration by GlyT1 at excitatory synapses within the locomotor circuitry, we show that GlyT1 antagonism results in an NMDAR-dependent increase in locomotor-burst frequency but not amplitude, replicating the effect of exogenous d-serine. The effect of GlyT1 blockade on network activity is therefore likely to result from the accumulation at excitatory synapses of glycine released at inhibitory synapses, as previously demonstrated (Ahmadi et al. 2003; Fern et al. 1996). Consistent with our results, inhibition of GlyT1 potentiates NMDAR currents in the brainstem (Lim et al. 2004) and enhances swimming in *Xenopus* tadpoles in a manner resembling application of glycine or d-serine (Issberner and Sillar 2007). Our data highlight the importance of GlyT1 in the gating of NMDARs and the regulation of rhythmic activity in the mouse spinal cord, a role that is conserved between amphibians and mammals (Issberner and Sillar 2007).

Modulation of GlyT1 activity to adjust the availability of glycine at excitatory synapses could provide a mechanism for the regulation of motor behaviors (Issberner and Sillar 2007). GlyT1 is modulated, for instance, by protein kinase C, which is activated by endogenous modulators of locomotor networks released during network activity, including 5-HT (Anji et al. 2001) and acetylcholine (Sculptoreanu et al. 2001). GlyT1 is also modulated by brain-derived neurotrophic factor (BDNF) (Aroeira et al. 2014), which is released in an activity-dependent manner (Goodman et al. 1996; Marini et al. 1998). Thus GlyT1 is equipped to mediate the dynamic regulation of glycine availability at excitatory synapses suggested by our data and may in this way confer flexibility on the output of spinal networks.

Given that glycine and d-serine appear to have opposite roles in determining locomotor frequency, activity-dependent changes in their availability may provide a mechanism for speed control and perhaps contribute to gait selection (Bellardita and Kiehn 2015; Talpalar and Kiehn 2010; Talpalar et al. 2013). Activity-dependent increases in d-serine availability would provide negative feedback onto the network to reduce locomotor speed, whereas increases in glycine would provide positive feedback to increase it. The existence of distinct mechanisms for regulating coagonist availability at NMDARs expressed by excitatory and inhibitory neurons may in this way permit glutamatergic signaling to be fine tuned, extending the range of outputs that can be generated by the locomotor CPG. Alternatively, simultaneous increases in the availability of facilitatory glycine and inhibitory d-serine may be important in stabilizing network output, ensuring smooth movement as speed changes. Changes in coagonist availability may also be important in nonrhythmic motor behaviors. For instance, an increase in coagonist availability resulting from sustained release of glutamate might be expected during slow, sustained motor behaviors in which muscle contraction is protracted, such as standing and grasping. During such activities, release of a coagonist to potentiate NMDAR currents could help to maintain activity in motoneurons.

It was beyond the scope of this study to consider the effects of glycine and d-serine on synaptic potentials recorded from individual neurons. Future studies should analyze the effects of glycine and d-serine on NMDAR currents recorded from specific populations of excitatory and inhibitory neurons and determine whether currents vary in response to local activity and fluctuations in glutamate release. In addition, this study did not consider which cell types are responsible for the regulation of coagonist availability. GlyT1 is preferentially expressed by astrocytes in the gray matter of the spinal cord and brainstem (Cubelos et al. 2005; Zafra et al. 1995a, 1995b), and astrocytes are required for the synthesis (Wolosker 2011; Ehmsen et al. 2013), degradation (Sasabe et al. 2014; Verrall et al. 2007) and, at some synapses, secretion (Henneberger et al. 2010; Mothet et al. 2005; Verrall et al. 2010; Yang et al. 2003) of d-serine. Furthermore, proper regulation of the NMDAR coagonist binding site has been demonstrated to require functional interactions between neurons and astrocytes (Le Bail et al. 2015). Future studies should therefore address the relative contributions of astrocytes and neurons to the regulation of the coagonist binding site in spinal motor networks.

Regulation of NMDARs is essential to proper functioning of spinal locomotor networks. In healthy animals, the coagonist binding site may provide a mechanism by which glutamatergic signaling is refined, permitting network activity to be adjusted as required. By contrast, dysregulation of NMDARs in the spinal cord may result in excitotoxicity (Shleper et al. 2005) and is implicated in the pathogenesis of ALS (Paul et al. 2014; Sasabe et al. 2007). This study provides the first evidence that NMDARs in the spinal cord are regulated by both d-serine and glycine acting at distinct populations of NMDARs, and that coagonist availability varies with neuronal activity. Furthermore, GlyT1 is shown to control the availability of glycine at excitatory synapses. Together, these findings reveal previously unexplored complexity in glutamatergic signaling during activity of mammalian locomotor networks.

## GRANTS

This work was supported by an Institutional Strategic Support Fund grant from the Wellcome Trust.

## DISCLOSURES

No conflicts of interest, financial or otherwise, are declared by the authors.

## AUTHOR CONTRIBUTIONS

D.A. and G.B.M. conceived and designed research; D.A. performed experiments; D.A. analyzed data; D.A. and G.B.M. interpreted results of experiments; D.A. prepared figures; D.A. drafted manuscript; D.A. and G.B.M. edited and revised manuscript; D.A. and G.B.M. approved final version of manuscript.
